# Recovery of Five Complete Influenza A(H1N1)pdm09 Genome Sequences from the 2015 Influenza Outbreak in India by Metagenomic Sequencing

**DOI:** 10.1128/genomeA.00511-18

**Published:** 2018-06-28

**Authors:** Paban Kumar Dash, Chitra Pattabiraman, Kundan Tandel, Shashi Sharma, Jyoti S. Kumar, Shilpa Siddappa, Malali Gowda, Sudhir Krishna, Manmohan Parida

**Affiliations:** a Division of Virology, Defence Research and Development Establishment (DRDE), Gwalior, India; b National Centre for Biological Sciences, TIFR, Bangalore, India; c Next-Generation Sequencing Facility, Centre for Cellular and Molecular Platforms, Bangalore, India

## Abstract

Five complete (H1N1)pdm09 viral sequences were recovered from hospitalized individuals during the 2015 influenza outbreak by metagenomic sequencing. Four of the genomes are from oropharyngeal swabs, and one is from an isolate. All five sequences belong to an emerging 6B clade. Studying them further is critical for outbreak preparedness.

## Abstract (precis)

Five complete (H1N1)pdm09 viral sequences were recovered from hospitalized individuals during the 2015 influenza outbreak by metagenomic sequencing. Four of the genomes are from oropharyngeal swabs, and one is from an isolate.

## GENOME ANNOUNCEMENT

Influenza A(H1N1)pdm2009 viruses cause swine flu in humans (1). These RNA viruses are known to acquire mutations rapidly. Mapping the mutations through time is critical for tracing the emergence of drug resistance, predicting the emergence of a pandemic strain, and evaluating the effectiveness of the vaccine (2). In 2015, there was a particularly severe influenza outbreak in India, but very few complete H1N1 genomes from this outbreak are presently available (3).

Acute-phase respiratory swab (nasopharyngeal/throat/nasal) samples from patients suspected of having an influenza A(H1N1)pdm09 infection were collected by the district health administrators of Madhya Pradesh, India. These were referred to the Defence Research and Development Establishment (DRDE), Gwalior, India, a nodal reference lab for pandemic H1N1 swine flu strain molecular diagnosis. WHO guidelines were followed for sample collection and transport and laboratory investigation in a biosafety level 3 laboratory. We sequenced the RNA from oropharygeal swabs of patients that tested positive for H1N1 by PCR.

Six clinical samples, three cell culture isolates, and one healthy control were subjected to metagenomic/unbiased sequencing on the Ion Proton/PGM sequencing platform. RNA was extracted using the QIAamp viral RNA minikit (Qiagen, Germany), and we prepared the sequencing libraries using the Ion Proton library preparation kit (Ion Total RNA-Seq kit V2). Samples were multiplexed in sets of 5 to 6 using an Ion Xpress RNA-Seq barcode kit. Template preparation was carried out using an Ion PI HI-Q template OT2 200 kit. We sequenced 8 pmol of the pooled libraries using the Ion PI HI-Q 200 kit and preprimed chips (Ion PI chip kit V3).

Sequencing reads were demultiplexed, and the resulting FASTQ files were used for the analysis. The SNAP (snap-1.0 beta.16) alignment tool (4) was used to remove host sequences (human genome and mRNA for samples and canine genome for isolates) and rRNA sequences (from the SILVA database [5]). Mapping assembly was performed using the MIRA 4.0.2 tool (6) with influenza A/California/07/2009(H1N1) as the reference strain. Genome editing was carried out using Gap5 (staden-2.0.0b11-2016) (7). The resulting consensus sequences were deposited in GenBank.

Concatenated and individual gene segment-wise phylogenetic analyses were carried out using the maximum likelihood algorithm employing the GTR + G + I nucleotide substitution model in the MEGA5 software program (8) and revealed the sequences to belong to an emerging 6B clade.

Changes in these strains, with reference to the pandemic strain, are important to study in order to understand the evolution of the virus and emergence of drug resistance and to prepare for outbreaks.

### Accession number(s).

The five viral genome sequences reported here were deposited in GenBank under the accession numbers KX078482 to KX078489, KX078490 to KX078497, KX078498 to KX078505, KX078506 to KX078513, and KX078514 to KX078521.
